# Maternal Sevoflurane Exposure Causes Abnormal Development of Fetal Prefrontal Cortex and Induces Cognitive Dysfunction in Offspring

**DOI:** 10.1155/2017/6158468

**Published:** 2017-09-25

**Authors:** Ruixue Song, Xiaomin Ling, Mengyuan Peng, Zhanggang Xue, Jing Cang, Fang Fang

**Affiliations:** Department of Anesthesiology, Zhongshan Hospital, Fudan University, 180 Fenglin Rd, Shanghai 200032, China

## Abstract

Maternal sevoflurane exposure during pregnancy is associated with increased risk for behavioral deficits in offspring. Several studies indicated that neurogenesis abnormality may be responsible for the sevoflurane-induced neurotoxicity, but the concrete impact of sevoflurane on fetal brain development remains poorly understood. We aimed to investigate whether maternal sevoflurane exposure caused learning and memory impairment in offspring through inducing abnormal development of the fetal prefrontal cortex (PFC). Pregnant mice at gestational day 15.5 received 2.5% sevoflurane for 6 h. Learning function of the offspring was evaluated with the Morris water maze test at postnatal day 30. Brain tissues of fetal mice were subjected to immunofluorescence staining to assess differentiation, proliferation, and cell cycle dynamics of the fetal PFC. We found that maternal sevoflurane anesthesia impaired learning ability in offspring through inhibiting deep-layer immature neuron output and neuronal progenitor replication. With the assessment of cell cycle dynamics, we established that these effects were mediated through cell cycle arrest in neural progenitors. Our research has provided insights into the cell cycle-related mechanisms by which maternal sevoflurane exposure can induce neurodevelopmental abnormalities and learning dysfunction and appeals people to consider the neurotoxicity of anesthetics when considering the benefits and risks of nonobstetric surgical procedures.

## 1. Introduction

Advances in prenatal imaging and innovations in surgical techniques have resulted in a wide range of fetal interventions [1]. Because of the relatively long duration of such procedures and the necessity of general anesthesia, long-time inhalation of anesthetic such as sevoflurane is administered to help uterine quiescence and lower the premature birth risk. However, inhalation anesthetics could be powerful regulators of brain development and have been reported to contribute to detrimental behavioral deficits [2]. Several large cohort studies have investigated the neurotoxicity of anesthesia to the developing brain [3–5], but the data remain elusive. Recently, the “Drug Safety Communication” has issued a warning that general anesthesia used in pregnant women in their third trimester may affect the development of the children's brain [6]. Sevoflurane is one of the most prevalent inhalation anesthetics in nonobstetric surgeries. Although sevoflurane has smaller potency to cause neurotoxicity to the developing brain compared with other general anesthetic such as isoflurane [7], there were still some preclinical studies reported that sevoflurane could cause neurological deficits [8, 9]. While neurogenesis abnormality is thought to play a vital role [10–12], the concrete impact of sevoflurane on fetal brain development remains poorly understood.

Most studies on the sevoflurane-induced neurotoxicity have focused on the change in the development of the hippocampus [10, 13]. It is worth noting that the third trimester is a stage at which there are high levels of neurogenesis throughout the cortex and that the development of the prefrontal cortex (PFC), a seat of the highest-order cognitive functions, plays critical roles in the onset and development of many neurodevelopmental deficits [14]. Three main types of neural progenitors, neural stem cell, radial glial cell, and intermediate progenitor cell, have been identified to be involved in the proliferation and differentiation of the PFC [15]. The neurogenesis of the PFC is accomplished by a regular production and migration of neurons in a deep to superficial order [16]. Former *in vitro* studies have shown that self-renewal capacity and the subsequent differentiation of neural progenitors could be disturbed by sevoflurane [12, 17]. Our earlier study has also shown a significant proliferation inhibition in neural progenitors after sevoflurane exposure [10]. Cell cycle dynamics, including the progression and exit of cell cycle, is important in cell fate decisions during neurogenesis [18]. Our former study has found that sevoflurane could lead to postoperative cognitive dysfunction in aged mice through interfering cell cycle dynamics in neurons [19].

All of the knowledge mentioned above prompted us to determine whether the sevoflurane-induced neurotoxicity could be attributed to the cell cycle-related abnormality in the development of the fetal PFC. Thus, we hypothesized that maternal sevoflurane exposure may disturb the differentiation and proliferation of neural progenitors by interfering the cell cycle dynamics, which finally lead to learning deficits in offspring. Our results demonstrated that maternal sevoflurane exposure induced cell cycle arrest in neural progenitors of the fetal PFC, lead to decrease in neuronal output and inhibition in neural progenitor replication, and finally resulted in learning deficits in offspring.

## 2. Materials and Methods

### 2.1. Mice Anesthesia

All procedures were approved by the Animal Care and Use Committee of Fudan University and followed institutional guidelines. Four-month old C57BL/6J female mice were mated with four-month old C57BL/6J male mice, and the pregnant mice were housed individually after identified. All of the animals were raised in a temperature-controlled (22°–23°C) room under a 12 h light/dark period; water and standard mouse chow were available ad libitum. The pregnant mice were randomly assigned to a control group or a sevoflurane group at gestational day 15.5 (G15.5). Pregnant mice in the sevoflurane group received 2.5% sevoflurane in 100% oxygen for 6 h in an anesthetizing box, while the pregnant mice in the control group received 100% oxygen for 6 h. The size of the anesthetizing box was 20 × 20 × 20 cm^3^. The gas flow rate was 2 L/min in the first 5 min for induction and then 1 L/min for maintenance. The concentrations of sevoflurane and oxygen were continuously monitored with a gas analyzer (Drager Inc.). Sevoflurane anesthesia was discontinued by terminating sevoflurane supply. The mortality rate was <1% in the present study.

### 2.2. Morris Water Maze (MWM) Test

The MWM test was performed as described in our former study [10]. For grouping, the male offspring at postnatal day 30 were delivered to the same group as their mothers. Specifically, the offspring were tested in the MWM four trials per day for five consecutive days (from P30 to P34). Each mouse was given 60 s to search the platform. The platform was then removed at P35, and the mice were placed in the opposite quadrant to swim for 60 s. The swimming speed, escape latency, platform crossing times, and the percentage of time target quadrant were recorded with a video tracking system (Shanghai Jiliang Software Technology Co. Ltd., China). All the mice were dried under a heat lamp for 5–8 min after each trail.

### 2.3. Measurement of Proliferation, Cell Cycle Exit, and S-Phase Duration of Neural Progenitors with Bromodeoxyuridine (BrdU) and Iododeoxyuridine (IdU)

For the determination of proliferation, pregnant mice were injected i.p. with a single BrdU (Sigma, B5002) dose (50 mg/kg of body weight) at the start of experiment and the pregnant mice were sacrificed 6 h later (at the end of the sevoflurane/oxygen exposure). The percentage of proliferating cells was calculated as BrdU^+^/DAPI [10]. For cell cycle exit assay, the pregnant mice were also injected i.p. with the same dose of BrdU at the start of experiment but sacrificed 18 h later. Cortices from embryos that had been labelled with BrdU for 18 h were visualized for both Ki67 reactivity and BrdU incorporation. Those neural progenitors that had divided in the previous 18 h and subsequently exited the cell cycle would have taken up BrdU but would not stain for Ki67; therefore, the evaluation of BrdU^+^Ki67^−^/total BrdU^+^ can serve as an effective detection method for cell cycle exit [20]. To determine the S-phase duration, pregnant mice at G15.5 were injected i.p. with an IdU (Sigma, I7125) dose (50 mg/kg of body weight) 4 h after the start of the sevoflurane/oxygen exposure, followed by BrdU injection (50 mg/kg of body weight) 1.5 h later. The pregnant mice were then killed at the end of the 6 h sevoflurane/oxygen exposure (0.5 h after BrdU injection), and the embryos were processed to immunofluorescence to reveal IdU/BrdU. The length of S-phase (*T*_s_) was calculated with the following paradigm described by Quinn et al. [21]: the number of cells labeled with IdU but not BrdU was regarded as *L*_cells_, referring to the cells that have taken up IdU but left S-phase and failed to take up BrdU during the interval between IdU and BrdU injection (*T*_i_ = 1.5 h). The number of cells labeled with BrdU is designated *S*_cells_. Then *T*_s_ can be calculated with the following formula: *T*_s_/*T*_i_ = *S*_cells_/*L*_cells_.

### 2.4. Immunofluorescence

A cesarean section was performed to extract the embryos, and the fetal brains were then fixed overnight in 4% paraformaldehyde. For cryosectioning, fixed brains were equilibrated in 20% (wt/vol) sucrose in PBS followed by 30% sucrose in PBS overnight at 4°C. Brains were then embedded with Tissue-TEK (O.C.T., Sakura Finetek) and cryosectioned at 12 *μ*m. For immunofluorescence, the cryosections were first washed with PBS and then incubated with blocking solution (10% goat serum in PBS, 0.03% Triton X-100) for 2 h at 37°C. Sections were next incubated with primary antibodies diluted in blocking solution overnight at 4°C. The tissues were then washed in PBS and incubated with appropriate secondary antibodies for 1 h at room temperature. Cell nucleus was counterstained with DAPI (Sigma, 1 : 1000). For BrdU and IdU detection, an additional antigen retrieval step was performed before blocking by using HCl (2 N HCl, 15 min incubation at 37°C) [10]. The following primary antibodies were used: Tbr1 (Abcam, ab31940, 1 : 200), Satb2 (Abcam, ab51502, 1 : 200), NeuN (Abcam, ab104224, 1 : 200), GFAP (Abcam, ab10062, 1 : 200), BrdU only (Abcam, ab6326, 1 : 1000), Ki67 (Abcam, ab16667, 1 : 500), caspase-3 (Abcam, ab13847, 1 : 200), nestin (Abcam, ab6142, 1 : 200), Pax6 (Abcam, ab5790, 1 : 200), Tbr2 (Abcam, ab23345, 1 : 200), BrdU and IdU (BD Biosciences, 347580, 1 : 200), Ccnd1 (Abcam, ab6134175, 1 : 200), and PH3 (Abcam, ab5176, 1 : 200). Secondary antibodies used were goat anti-mouse Alexa Fluor 488, goat anti-mouse Alexa Fluor 594, goat anti-rat Alexa Fluor 594, and goat anti-rabbit Alexa Fluor 488 (all from Abcam, diluted at 1 : 200).

### 2.5. Image and Cell Count

Immunofluorescence analysis was performed on data collected from cortexes of at least 3 (*n* ≥ 3) embryos of each group. Fluorescence images were acquired using a Leica TCS SP2 confocal microscope, and all images showing the target parameters for the control group versus the sevoflurane group were acquired with the same settings during each microscope session. Cells were counted in four 100 *μ*m-wide strips through the prefrontal cortex, in a minimum of three nonadjacent sections from each embryo, with the image J pro plus software.

### 2.6. Statistical Analysis

Values are presented as means ± SEM. Two-way ANOVA with repeated measurements was used to analyze the difference of escape latency in the MWM test, and the Bonferroni method was used to adjust the multiple comparisons. Two-tailed Student's *t*-test was performed for statistical evaluation of immunofluorescence. *P* values less than 0.05 were considered statistically significant.

## 3. Results

### 3.1. Maternal Sevoflurane Exposure Impaired Spatial Learning and Memory Ability in Offspring

**Figure 1:**
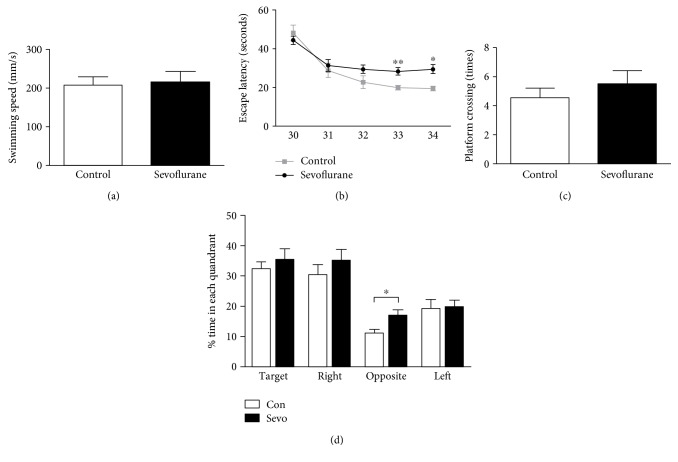
Maternal sevoflurane exposure impaired learning and memory ability in offspring. (a) No significant differences in swimming speed were found between the control group and the sevoflurane group. (b) The escape latency of MWM in the sevoflurane group was longer than that in the control group. (c) No significant differences in platform crossing times were found between the control group and the sevoflurane group. (d) The %time in the opposite quadrant in the sevoflurane group was longer than that in the control group. Data are expressed as the mean ± SEM. ^∗^*P* < 0.05 and ^∗∗^*P* < 0.01.

All of the pregnant mice delivered offspring at G20.5–G22.5, and the offspring were reared for 30 days before being assigned to the MWM test. There was no statistical significance in the swimming speed between the control group and the sevoflurane group (Figure 1(a)) (*n* = 9, *F* = 1.590, and *P* = 0.525), which excluded the possibility that the learning changes observed in the current study were influenced by sensorimotor disturbances. A two-way ANOVA with repeated measurement on the escape latency (the time that each mouse took to reach the platform) revealed a statistical interaction between the time and the group (Figure 1(b)) (*n* = 9, *F* = 7.740, and *P* = 0.013) in the cued trials. Specially, the offspring in the sevoflurane group had significantly longer escape latency compared with those in the control group at P33 and P34 (Figure 1(b)) (*P* = 0.001 and 0.013, resp.). Moreover, we found that %time in the opposite quadrant was longer in the sevoflurane group compared to the control group in the probe test at P35 (*n* = 9, *F* = 2.417, *P* = 0.025). However, we found no significant difference in platform crossing times (Figure 1(c)) (*n* = 9, *F* = 2.250, and *P* = 0.423). These data indicated that maternal sevoflurane exposure impaired spatial learning and memory ability in offspring.

### 3.2. Sevoflurane Decreased the Production of Deep-Layer Immature Neurons in the Fetal PFC

**Figure 2:**
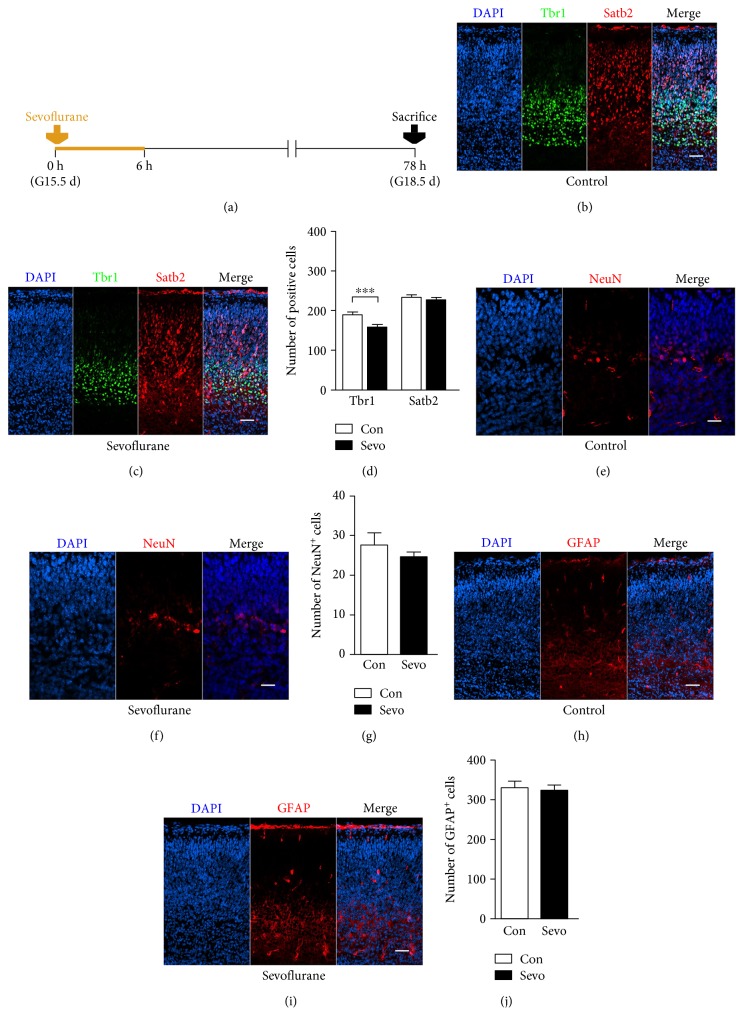
Maternal sevoflurane exposure decreased the production of deep-layer immature neurons. (a) Schematic diagram of the timing of sevoflurane exposure and sacrifice to assess the differentiation in the fetal PFC. (b, c) Tbr1 (green) and Satb2 (red) immunofluorescence, combined with DAPI staining (blue) in the cortical plate at G18.5. Scale bars, 20 *μ*m. (d) Quantification of the Tbr1^+^ and Satb2^+^ cells of the control and sevoflurane groups. (e, f) NeuN (red) immunofluorescence and DAPI staining (blue) in the cortical plate at G18.5. Scale bars, 20 *μ*m. (g) Quantification of the NeuN^+^ cells of the control and sevoflurane groups. (h, i) GFAP (red) immunofluorescence and DAPI staining (blue) in the cortical plate at G18.5. Scale bars, 20 *μ*m. (j) Quantification of the GFAP^+^ cells of the control and sevoflurane groups. Data are expressed as the mean ± SEM. ^∗∗∗^*P* < 0.001.

To investigate whether sevoflurane disturbs embryonic brain development, we first examined the numbers of neural cells in the fetal PFC. To distinguish between the upper-layer and deep-layer newborn neurons, we used the established Tbr1 to label layers V−VI immature neurons and Satb2 to label layers II–IV immature neurons [22]. We also selected NeuN and GFAP to identify mature neurons [23] and mature astrocytes [20], respectively. Tbr1^+^ immature neurons in the fetal PFC were significantly decreased after sevoflurane exposure (Figures 2(b), 2(c), and 2(d)) (*n* = 15, *F* = 1.369, and *P* = 0.001) while there were no significant differences in the numbers of Satb2^+^ immature neurons (Figures 2(b), 2(c), and 2(d)) (*n* = 15, *F* = 1.106, and *P* = 0.789), NeuN^+^ mature neurons (Figures 2(e), 2(f), and 2(g)) (*n* = 8, *F* = 6.278, and *P* = 0.406), and GFAP^+^ mature astrocytes (Figures 2(h), 2(i), and 2(j)) (*n* = 6, *F* = 1.656, and *P* = 0.796). Together, these data showed that maternal sevoflurane exposure decreased the generation of deep-layer immature neurons.

### 3.3. Sevoflurane Suppressed the Proliferation of the Fetal PFC

**Figure 3:**
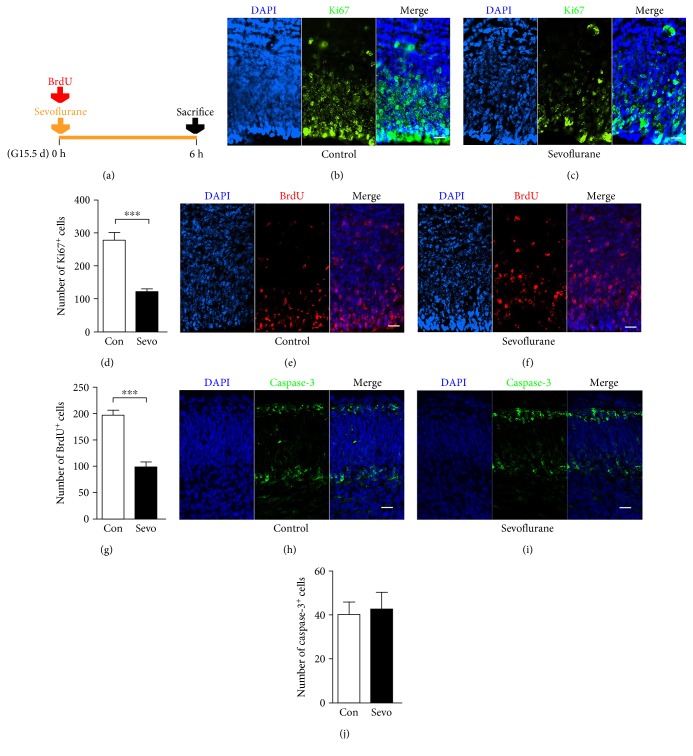
Maternal sevoflurane exposure suppressed the proliferation of the fetal PFC. (a) Schematic diagram of the timing of sevoflurane exposure, BrdU injection, and sacrifice to assess the proliferation and apotosis of the fetal PFC. (b, c) Ki67 (green) immunofluorescence and DAPI staining (blue) in the cortical plate at G15.5. Scale bars, 20 *μ*m. (d) Quantification of the Ki67^+^ cells of the control and sevoflurane groups. (e, f) BrdU (red) immunofluorescence and DAPI staining (blue) in the cortical plate at G15.5. Scale bars, 20 *μ*m. (g) Quantification of the BrdU^+^ cells of the control and sevoflurane groups. (h, i) Caspase-3 (green) immunofluorescence and DAPI staining (blue) in the cortical plate at G15.5. Scale bars, 20 *μ*m. (j) Quantification of the GFAP^+^ cells of the control and sevoflurane groups. Data are expressed as the mean ± SEM. ^∗∗∗^*P* < 0.001.

Reduced numbers of deep-layer Tbr1^+^ immature neurons could result from reduced proliferation or increased apoptosis of the fetal PFC, so we next investigated the influence of sevoflurane on these processes. The fetal PFC of the sevoflurane group showed reduced numbers of Ki67^+^ (Figures 3(b), 3(c), and 3(d)) (*n* = 6, *F* = 7.115, and *P* = 0.0001) and BrdU^+^ neural cells (Figures 3(e), 3(f), and 3(g)) (*n* = 6, *F* = 1.111, and *P* = 0.0001), emphasizing the inhibition of proliferation. However, we have not detected any differences in the number of cells undergoing apoptosis between the two groups as judged by staining with caspase-3 (Figures 3(h), 3(i), and 3(j)) (*n* = 5, *F* = 1.817, and *P* = 0.809).

### 3.4. Sevoflurane Inhibited the Expansion of Neural Progenitors in the Fetal PFC

**Figure 4:**
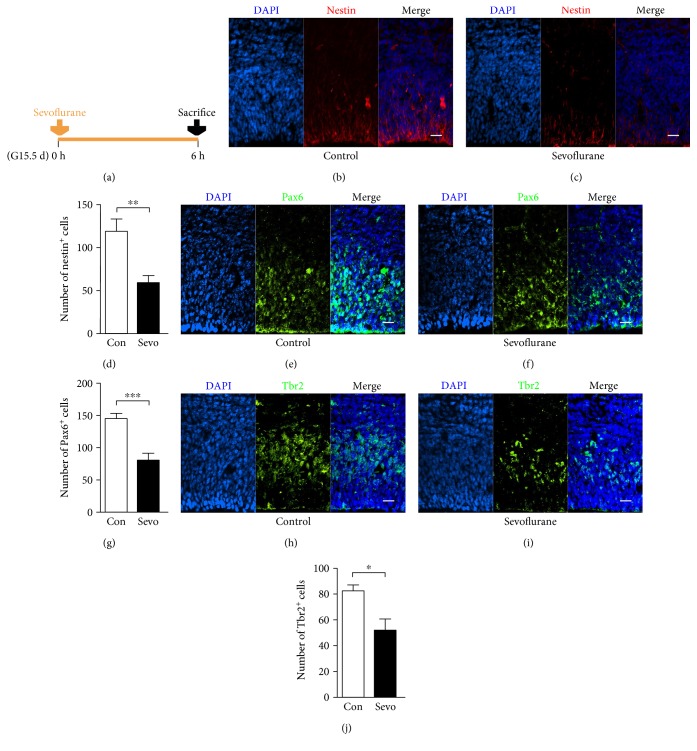
Maternal sevoflurane exposure inhibited the expansion of neural progenitors in the fetal PFC. (a) Schematic diagram of the timing of sevoflurane exposure and sacrifice to assess the abundance of neural progenitors. (b, c) Nestin (red) immunofluorescence and DAPI staining (blue) in the cortical plate at G15.5. Scale bars, 20 *μ*m. (d) Quantification of the nestin^+^ cells of the control and sevoflurane groups. (e, f) Pax6 (green) immunofluorescence and DAPI staining (blue) in the cortical plate at G15.5. Scale bars, 20 *μ*m. (g) Quantification of the Pax6^+^ cells of the control and sevoflurane groups. (h, i) Tbr2 (green) immunofluorescence and DAPI staining (blue) in the cortical plate at G15.5. Scale bars, 20 *μ*m. (j) Quantification of the Tbr2^+^ cells of the control and sevoflurane groups. Data are expressed as the mean ± SEM. ^∗^*P* < 0.05, ^∗∗^*P* < 0.01, and ^∗∗∗^*P* < 0.001.

Reduced labeling index of Ki67 and BrdU in the PFC suggested a decrease of neural progenitor pool after sevoflurane exposure, so we further performed nestin, Pax6, and Tbr2 immunostaining to label neural stem cell, radial glial cell, and intermediate progenitor cell, respectively [15]. We found a weak and sparse staining of nestin (Figures 4(b), 4(c), and 4(d)) (*n* = 8, *F* = 2.728, and *P* = 0.003), Pax6 (Figures 4(e), 4(f), and 4(g)) (*n* = 6, *F* = 1.649, and *P* = 0.001), and Tbr2 in the sevoflurane group (Figures 4(h), 4(i), and 4(j)) (*n* = 4, *F* = 3.209, and *P* = 0.025), which indicated that the neural progenitor abundance in the fetal PFC was inhibited after maternal sevoflurane exposure.

### 3.5. Sevoflurane Decreased Cell Cycle Exit and Increased S-Phase Duration of Neural Progenitors in the Fetal PFC

**Figure 5:**
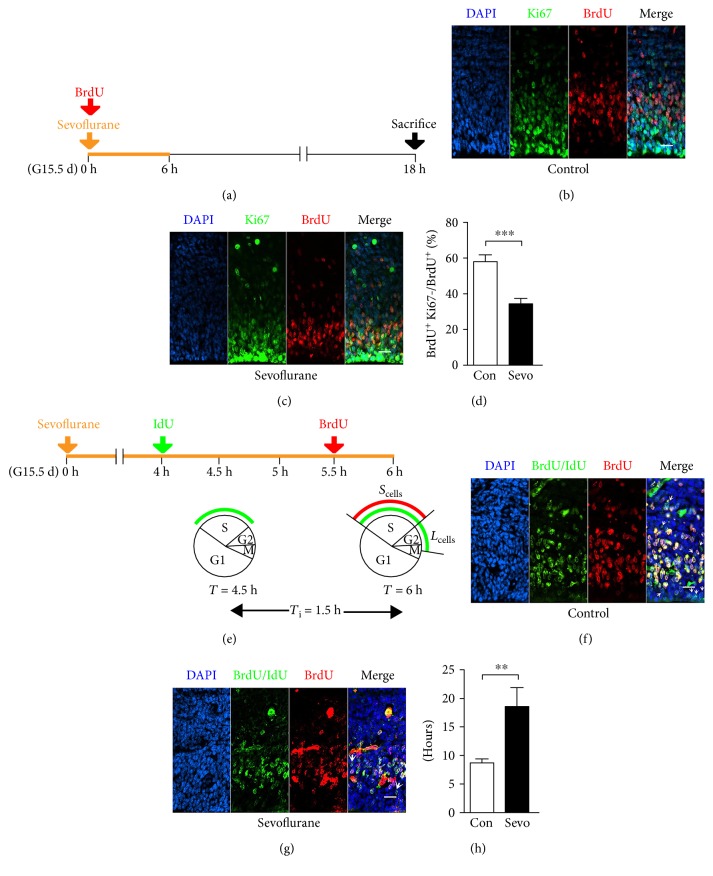
Maternal sevoflurane exposure decreased cell cycle exit and increased S-phase duration of neural progenitors in the fetal PFC. (a) Schematic diagram of the timing of sevoflurane exposure, BrdU injection, and sacrifice to assess the proportion of the cell cycle exit. (b, c) Coronal sections of the PFC from G16.5 mice were immunostained for BrdU (red) and Ki67 (green) at G16.5. Scale bars, 20 *μ*m. (d) Numbers of BrdU^+^Ki67^−^ cells are expressed as the numbers of BrdU^+^ cells. (e) Schematic diagram of the timing of sevoflurane exposure, IdU injection, and BrdU administration to assess the S-phase duration. *S*_cells_ = cells labeled with BrdU; *L*_cells_ = cells labeled with IdU but not BrdU. (f, g) Coronal section through the cortex of the G15.5 fetal brain immunostained with antibodies specific for both BrdU and IdU (green) and BrdU alone (red) to identify *L*_cells_ (green-only cells) and *S*_cells_ (red and green double-labeled cells). Arrowheads indicate *L*_cells_. Scale bars, 20 *μ*m. (h) Quantification of the length of S-phase in G15.5 embryos. Data are expressed as the mean ± SEM. ^∗∗^*P* < 0.01 and ^∗∗∗^*P* < 0.001.

Since cell cycle dynamics could affect the proliferation and differentiation of the developing brain [24], we postulated sevoflurane-induced reduction in neural progenitor proliferation and newborn neuron production could reflect cell cycle dysregulation. With the assessment of cell cycle exit, we found that the fetal PFCs in the sevoflurane group contained significantly less Ki67-negative neural progenitors that had incorporated BrdU in the previous 18 h (Figures 5(b), 5(c), and 5(d)) (*n* = 13, *F* = 1.029, and *P* = 0.0001). Moreover, our double-labeling experiments of IdU/BrdU revealed a significant increase in the S-phase duration after sevoflurane exposure (Figures 5(f), 5(g), and 5(h)) (*n* = 15, *F* = 19.63, and *P* = 0.004). Taken together, our data indicated that sevoflurane decreased cell cycle exit and increased the S-phase duration of neural progenitors in the fetal PFC.

### 3.6. Sevoflurane Did Not Influence the Duration of G1-, M-, and G2-Phases of Neural Progenitors in the Fetal PFC

**Figure 6:**
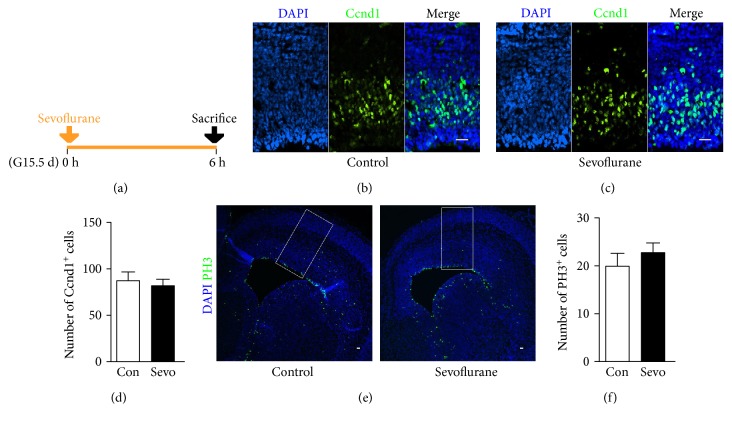
Maternal sevoflurane exposure did not influence the duration of G1-, M-, and G2-phases of neural progenitors in the fetal PFC. (a) Schematic diagram of the timing of sevoflurane exposure and sacrifice to assess the expression of Ccnd1 and PH3. (b, c) Ccnd1 (green) immunofluorescence and DAPI staining (blue) in the cortical plate at G15.5. Scale bars, 20 *μ*m. (d) Quantification of the Ccnd1^+^ cells of the control and sevoflurane groups. (e) PH3 (green) immunofluorescence and DAPI staining (blue) in the cortical plate at G15.5. PH3-positive cells were counted in radially arranged 200 mm-wide boxes, as illustrated. Scale bars, 20 *μ*m. (f) Quantification of the PH3^+^ cells of the control and sevoflurane groups. Data are expressed as the mean ± SEM.

In addition to cell cycle exit and S-phase duration, the dysregulation of G1, M, and G2 may also alter cell fate in the developing brain [25]. In the current study, no significant differences were found between the control group and the sevoflurane group when analyzing the proportion of neural progenitors that expressed the Ccnd1, a cyclin expressing from mid-G1 to late G1 (Figures 6(b), 6(c), and 6(d)) (*n* = 8, *F* = 2.073, and *P* = 0.670). PH3, a specific indicator of late G2- and M-phases, has been used to investigate the duration of G2- and M-phases in neural progenitors [26, 27]. We did not found any significant difference in the expression of PH3 (Figures 6(e) and 6(f)) (*n* = 10, *F* = 1.744, and *P* = 0.420). These data have indicated that sevoflurane did not influence the duration of G1-, M-, and G2-phases of neural progenitors in the fetal PFC.

## 4. Discussion

In this study, we evaluated the *in vivo* toxic effects of maternal sevoflurane exposure via investigating the learning ability of offspring and the development of the fetal PFC. Our data indicated that maternal gestational exposure to sevoflurane was associated with increased risk for learning deficits in offspring. The sevoflurane neurotoxicity may be due to the decrease in cell cycle exit and increase in the S-phase duration of neural progenitors, which consequently lead to proliferation inhibition and differentiation abnormality in the fetal PFC.

Anesthetics can be toxic to brain development, and the vulnerability mainly depends on three factors: the stage of brain development and the concentration and duration of the exposure [28]. In human, maternal and fetal procedures are usually performed in the second or early third trimester, a critical time for the proliferation and differentiation of the fetal brain [29]. From a developmental perspective, these processes in rodents, unlike human beings, begin from the middle of the second trimester and continues to the time of birth [29]. In the present study, we chosen the pregnant mice in early third trimester (G15.5) to study the neurotoxicity of sevoflurane. While low concentration of sevoflurane such as 1.5% has been reported to do no harm to the brain development [30], our previous study [10] has found 2.5% sevoflurane could lead to learning deficits in offspring. Additionally, a prolonged exposure of sevoflurane such as 6 h has been reported to suppress the proliferation of neural progenitors [17] and caused learning impairments in offspring [8]. Therefore, the pregnant mice of the sevoflurane group in the current study were exposed to 2.5% sevoflurane for 6 h to study the neurodevelopmental change of the fetal brain.

The Morris water maze is a reliable method for assessing the ability of learning and memory [31]. In this test, spatial learning ability is determined by the escape latency in cued trials and reference memory is assessed with preference for the platform area in the probe test [31]. We have found a significant increase of the averaged escape latency in the offspring of the sevoflurane group (Figure 1(b)), indicating the impairment in spatial learning ability after prenatal sevoflurane exposure. In analyzing the probe test, we did not find any significant differences in the platform crossing times and the %time in the target, right and left adjacent quadrants (Figures 1(c) and 1(d)). However, we have found that the %time in the opposite quadrant was longer in the sevoflurane group than in the control group (Figure 1(d)), indicating that maternal sevoflurane exposure could lead to memory impairment in the offspring. Our finding was consistent with prior studies reporting that sevoflurane anesthesia used in pregnant mice could affect the cognitive function in offspring [8, 10].

Learning is a highly dynamic process, and the PFC has been reported to play vital roles in this process [32]. Some mental diseases with symptoms of cognitive dysregulation are usually contributed to structural and pathophysiological abnormalities in the PFC [33]. A fundamental feature of fetal brain neurogenesis is that the positioning of neurons into vertical arrays specifies their functions [34]. As neurogenesis proceeds, newborn neurons migrate radially from the proliferative zone, past neurons generated earlier, settle in more outer layers, and finally form a six-layered cortex [34]. Upper layers (layers II–IV) are composed of late-born neurons while deep layers (layers V-VI) are of early-born neurons [35]. Dysregulation in the neurogenesis of the prefrontal cortex, such as incomplete clustering and abundance of newly generated neurons, has been reported in neurological disorders [36]. In the present study, the production of immature deep-layer neurons in the PFC, identified by Tbr1, was significantly inhibited after sevoflurane exposure (Figures 2(b), 2(c), and 2(d)). Tbr1 is a transcription factor necessary for directing immature neurons to a glutamatergic phenotype, and the downregulation of Tbr1^+^ neurons in the developing brain could lead to neurological disorders [37]. Therefore, the reduction in Tbr1^+^ neuron production may partly attribute to the learning impairment in offspring after sevoflurane exposure. Satb2 is involved in specifying callosal projection neurons [38], and the results of Satb2 staining (Figures 2(b), 2(c), and 2(d)) may suggest that the callosal connectivity of the fetal brain was not significantly affected by prenatal sevoflurane exposure. Moreover, we have not found any significant differences in the number of mature neurons (identified by NeuN staining) and mature astrocytes (identified by GFAP staining) in the PFC (Figures 2(e), 2(f), 2(g), 2(h), 2(i), and 2(j)). Given that neural progenitors give rise to different types of neurons and glial cells according to the intrinsic time course [39], the sevoflurane exposure time of G15.5 selected in our study may be just at the particular temporal window when the deep-layer immature neurons emerge.

The decrease in the production of deep-layer immature neurons in the PFC may be attributed to decreased neurogenesis and/or increased neurodegeneration. In the present study, we clearly showed that sevoflurane decreased the number of BrdU-labeled and Ki67-positive cells in the PFC of fetal mice (Figures 3(b), 3(c), 3(d), 3(e), 3(f), and 3(g)). This is consistent with a previously published report showing that the proliferation of cultured neural progenitors was decreased significantly after sevoflurane exposure [12]. Zheng et al. [8] have reported that sevoflurane activates caspase-3 in the total fetal brain, but we did not find any significant changes in the number of caspase-3^+^ cells in the PFC (Figures 3(h), 3(i), and 3(j)). The different findings of our study and Zheng's study may suggest that maternal sevoflurane exposure has different impacts on different regions of the fetal brain.

At the onset of neurogenesis, neural stem cells, the primary cortical stem cells, express nestin and undergo mitosis at the apical surface of the cortex. Radial glial cells are transformed from neural stem cells and express the transcription factor Pax6 [40, 41]. Radial glial cells also undergo divisions to generate intermediate progenitor cells, expressing Tbr2 [15, 41]. These neural progenitors repeatedly undergo self-renewal mitosis and form a progenitor pool at the apical surface of the cortex [15]. As we noticed that the proliferation inhibition mainly occurred in the apical surface of the cortex (Figures 3(b), 3(c), 3(d), 3(e), 3(f), and 3(g)), the region of neural progenitors, we supposed that maternal sevoflurane exposure might disturb the expansion of the neural progenitor pool. Therefore, we estimated the number of neural progenitor with immunofluorescence and found a significant decrease in the number of nestin^+^, Pax6^+^, and Tbr2^+^ cells (Figures 4(b), 4(c), 4(d), 4(e), 4(f), 4(g), 4(h), 4(i), and 4(j)). As the main source of PFC neurons, it is easy to deduce that this reduction in neural progenitor is one of the reasons leading to the decrease in the production of deep-layer immature neurons.

The proliferation and differentiation of neural progenitors are influenced by not only cell cycle exit but also cell cycle progression [40]. With the staining of BrdU and Ki67, we found a significant decrease in the proportion of cell cycle exit after sevoflurane exposure (Figures 5(b), 5(c), and 5(d)), indicating that the neural progenitors in the sevoflurane group are incapable of exiting cell cycle and differentiating to immature neurons. Both the decrease in the cell cycle exit and the downregulation of deep-layer neuron production (Figures 2(b), 2(c), and 2(d)) have indicated an abnormality in fetal brain differentiation. The length of S-phase has been reported to be the main cell cycle parameter associated with the proliferative behavior, and the self-renewal neural progenitors exhibited a relatively longer S-phase than that committed to neuron production [27]. We have found a significant increase in the S-phase duration of the neural progenitors exposed to sevoflurane (Figures 5(f), 5(g), and 5(h)), further indicating the downregulation of differentiation in the fetal PFC. The S-phase duration is the main time for neural progenitors to control the quality of replicated DNA [27], and sevoflurane has been reported to significantly increase DNA damage in rodents [42]. Therefore, our observation of prolonged S-phase may also indicate that DNA replication of neural progenitors in the sevoflurane group is abnormal, pending further study. Interestingly, the decreased cell cycle exit and increased S-phase duration were not accompanied by an expansion of the neural progenitor pool (Figure 4). This prompted us to think whether maternal sevoflurane exposure could lead to cell cycle arrest in neural progenitors. To verify our hypothesis, we further tested the change in other cell cycle parameters, including G1-, M-, and G2-phases. In a recent study, sevoflurane was reported to delay G1-phase and lead to cell cycle arrest in embryonic stem cells [43]. However, in our study of Ccnd1 labeling, the G1-phase of neural progenitors was not affected by prenatal sevoflurane exposure (Figures 6(b), 6(c), and 6(d)). It is reasonable that sevoflurane may affect G1-phase in different cell types through different mechanisms. Altered G2- and M-phase duration has been reported to directly alter cell fate in neural progenitors [44, 45], but the effect of sevoflurane on these two cell cycle phases of neural progenitors has not yet been studied in detail. In the present study, we did not find any significant differences in the expression of PH3 (Figures 6(e) and 6(f)), an indicator of M-phase and late G2-phase [26]. Together, the assessment of cell cycle exit and progression have indicated that maternal sevoflurane exposure could cause cell cycle arrest at S-phase in neural progenitors, which was associated with disrupted differentiation and proliferation of the fetal PFC.

Our findings indicated that the cell cycle disturbance of the neural progenitors in the fetal PFC contributed to aberrant proliferation and differentiation after maternal sevoflurane exposure, which may finally lead to the functional neurological impairments in adult offspring. Our study helped to understand the mechanism of postoperative neurological impairments after prenatal sevoflurane exposure and appealed people to consider the neurotoxicity of anesthetics when considering the benefits and risks of nonobstetric surgical procedures.
